# Adolescent and Young Adult Cancer Representation in Claims Data

**DOI:** 10.1001/jamanetworkopen.2025.3281

**Published:** 2025-04-04

**Authors:** Jacob N. Stein, Christopher Baggett, Jennifer L. Lund, Hannah C. Winslow, KyungSu Kim, Katherine E. Reeder-Hayes

**Affiliations:** 1 Division of Oncology, Department of Medicine, University of North Carolina School of Medicine, Chapel Hill; 2 Department of Epidemiology, University of North Carolina Gillings School of Public Health, Chapel Hill; 3 Lineberger Comprehensive Cancer Center, Chapel Hill, North Carolina

## Abstract

This cohort study evaluates the representation of adolescent and young adult patients with cancer in claims research based on continuous insurance coverage criteria.

## Introduction

Adolescents and young adults (AYAs) (aged 13-39 years) represent a growing segment of the cancer population who face distinct challenges in receiving care, leading to adverse treatment experiences and stagnant survival outcomes.^1^ Research on AYA outcomes and care delivery remains scarce.^2^ A crucial need exists for clinical studies on AYA care delivery to improve care experiences and outcomes, with claims-based data offering valuable data.

Gaps in health insurance are more common among AYAs than any other US age group,^3^ and AYAs without insurance face shorter survival.^4^ Insurance patterns for AYAs are not well documented, hampering use of claims for research. Inclusion in claims-based research often requires continuous enrollment starting months before assessment for preexisting comorbidities and health care use. We evaluated various enrollment criteria and compared the resulting study-eligible populations of AYAs with cancer for claims research.

## Methods

This retrospective cohort study used the Cancer Information and Population Health Resource (CIPHR), which links cancer registry and private and Medicaid payer data across North Carolina. Detailed database methods were described previously.^5^ The study was approved by the University of North Carolina Institutional Review Board with a waiver of written informed consent as the project involved a secondary data analysis. The study followed the STROBE reporting guideline.

We included individuals aged 13 to 39 years diagnosed with common cancer types in the CIPHR from 2004 to 2019 linked with insurance claims from 2003 to 2020 (Figure). We compared 2 scenarios of continuous enrollment: 2 months before through 12 months after cancer diagnosis and month of diagnosis through 12 months after. We compared sociodemographic characteristics and insurance types across scenarios. Self-reported race and ethnicity (Black, Hispanic, White, other) data were included as variables because they are sociodemographic factors with a known association with differences in US health insurance coverage. The statistical analysis was performed on March 12, 2024, using SAS, version 9.4 (SAS Institute Inc).

**Figure.**
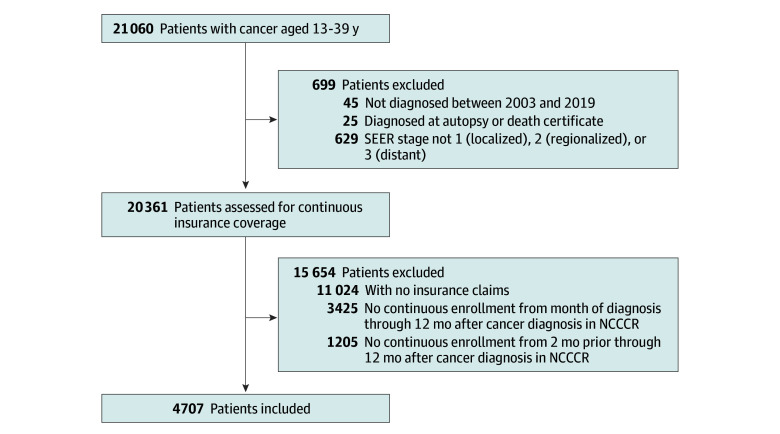
Flow Diagram for Study Cohort Inclusion NCCCR indicates North Carolina Central Cancer Registry; SEER, Surveillance, Epidemiology, and End Results.

## Results

We identified 20 361 AYAs from the cancer registry during the study period (median [IQR] age, 32 [26-36] years; 59.0% female and 41.0% male) (Table), of whom 54.1% did not match to any insurance claims. A total of 4707 patients (22.4%) had continuous enrollment from 2 months before to 12 months after their cancer diagnosis vs 5912 (28.1%) starting from month of diagnosis, which was driven by an increase in Medicaid-insured patients.

**Table.** Sociodemographic Characteristics of Adolescents and Young Adults in the State Cancer Registry and Across Claims Enrollment Criteria

Characteristic	Registry (N = 20 361), No. (%)	No claims (n = 11 024), No. (%)^b^	Continuous enrollment, No. (%)^a^					
			0 to 12 mo (n = 5912)			−2 to 12 mo (n = 4707)		
			Total	Private	Medicaid	Total	Private	Medicaid
Age at diagnosis								
Adolescent (13-19 y)	1793 (8.8)	847 (7.7)	646 (36.0)	173 (9.6)	473 (26.4)	544 (30.3)	167 (9.3)	377 (21.0)
Emerging adult (20-29 y)	5655 (27.8)	2932 (26.6)	1657 (29.3)	715 (12.6)	942 (16.7)	1291 (22.8)	650 (11.5)	641 (11.3)
Young adult (30-39 y)	12 913 (63.4)	7245 (65.7)	3609 (27.9)	1747 (13.5)	1862 (14.4)	2872 (22.2)	1617 (12.5)	1255 (9.7)
Sex								
Female	12 009 (59.0)	6356 (57.7)	3639 (30.3)	1548 (12.9)	2091 (17.4)	2933 (24.4)	1415 (11.8)	1518 (12.6)
Male	8348 (41.0)	4655 (43.3)	2272 (27.2)	1087 (13.0)	1185 (14.2)	1773 (21.2)	1019 (12.2)	754 (9.0)
Race and ethnicity								
Hispanic or Latino	1240 (6.1)	870 (7.9)	210 (16.9)	61 (4.9)	149 (12.0)	164 (13.2)	60 (4.8)	104 (8.4)
Non-Hispanic Black	3809 (18.7)	1520 (13.8)	1521 (39.9)	248 (6.5)	1273 (33.4)	1150 (30.2)	218 (5.7)	932 (24.5)
Non-Hispanic White	14 207 (69.8)	7983 (72.4)	3886 (27.4)	2201 (15.5)	1685 (11.9)	3153 (22.2)	2042 (14.4)	1111 (7.8)
Other^c^	1105 (5.4)	651 (5.9)	295 (26.7)	125 (11.3)	170 (15.4)	240 (21.7)	114 (10.3)	126 (11.4)
Cancer type								
Breast	5730 (28.1)	3094 (28.1)	1754 (30.6)	745 (13.0)	1009 (17.6)	1345 (23.5)	679 (11.8)	666 (11.6)
Leukemia	2357 (11.6)	1154 (10.5)	809 (34.3)	229 (9.7)	580 (24.6)	604 (25.6)	209 (8.9)	395 (16.8)
Lymphoma	4230 (20.8)	2064 (18.7)	1374 (32.5)	474 (11.2)	900 (21.3)	1064 (25.2)	436 (10.3)	628 (14.8)
Melanoma	3925 (19.3)	2429 (22.0)	858 (21.9)	644 (16.4)	214 (5.5)	779 (19.8)	604 (15.4)	175 (4.5)
Sarcoma	1697 (8.3)	849 (7.7)	532 (31.3)	193 (11.4)	339 (20.0)	429 (25.3)	181 (10.7)	248 (14.6)
Testicular (germ cell)	2422 (11.9)	1434 (13.0)	585 (24.2)	350 (14.5)	235 (9.7)	486 (20.1)	325 (13.4)	161 (6.6)
Location								
Urban	15 771 (77.5)	8875 (80.5)	4342 (27.5)	2069 (13.1)	2273 (14.4)	3512 (22.3)	1920 (12.2)	1592 (10.1)
Rural	4546 (22.3)	2119 (19.2)	1565 (34.4)	563 (12.4)	1002 (22.0)	1190 (26.2)	511 (11.2)	679 (14.9)

^a^ Percentages are of the specified category relative to the total registry cohort. For example, 30.6% of breast cancer registry cases were captured by continuous enrollment from 0 to 12 months after diagnosis; 13.0% of the breast cancer registry cases were among privately insured patients, while 17.6% were Medicaid insured.

^b^ Percentages of those with no claims are of that subpopulation.

^c^ Other race and ethnicity included American Indian or Alaska Native, Asian, and other race.

Less restrictive continuous enrollment criteria increased representation of Black patients from 30.2% to 39.9% of the total registry cohort, rural patients from 26.2% to 34.4%, and patients with leukemia from 30.2% to 34.3% and lymphoma from 25.2% to 32.5%. Clinical and demographic characteristics were similar across cohorts.

## Discussion

This cohort study found that less restrictive continuous enrollment criteria increased the number of AYAs with cancer eligible for inclusion in claims-based research while broadening the diversity, reflecting the processes involved in accessing health insurance for these patients. Many health systems assist patients with gaining insurance at the time of a cancer diagnosis, leading to an uptick in coverage in the month of diagnosis (1205 patients in our cohort). Notable differences were observed across insurers and eligibility scenarios. Rural and Black patients and patients with hematologic cancers were more likely have Medicaid.

A limitation of the less restrictive inclusion criteria is the lack of a lead-in period during which baseline comorbidities or prior care delivery can be assessed, acknowledging that comorbidities may be less common among AYAs. While conducted within a single state, North Carolina is representative of national demographics, and CIPHR reflects similar patterns as Surveillance, Epidemiology, and End Results and Medicare analyses.^6^

We found that AYAs with cancer are underrepresented in claims-based research and recommend that researchers use more lenient continuous enrollment criteria when studying AYAs through claims data to achieve a larger and more representative sample, which may lead to more generalizable findings. This approach is needed to better characterize the care for this underserved population.

## Notes

Supplement.
Data Sharing Statement
