# Supplementary material for: Adolescent and Young Adult Cancer Representation in Claims Data
Source: JAMA Netw Open. 2025 Apr 4;8(4):e253281. doi: 10.1001/jamanetworkopen.2025.3281 (PMC11971664; doi:10.1001/jamanetworkopen.2025.3281)
Supplement: Supplement. — Data Sharing Statement [file jamanetwopen-e253281-s001.pdf]

# Data Sharing Statement

Stein. Adolescent and Young Adult Cancer Representation in Claims Data. *JAMA Netw Open*. Published April 04, 2025. doi:10.1001/jamanetworkopen.2025.3281

**Data**
**Data available:** No

**Additional Information**
**Explanation for why data not available:** Interested researchers or regulatory bodies may reach out to the Cancer Information & Population Health Resource (CIPHR) for access to the data in de-identified format. As we are not the owners of the data, we are not permitted to share these data as per our existing data use agreements. We welcome inquiries from interested parties and can discuss on a case-by-case basis.
